# Identification of Small Molecule Inhibitors of *Pseudomonas aeruginosa* Exoenzyme S Using a Yeast Phenotypic Screen

**DOI:** 10.1371/journal.pgen.1000005

**Published:** 2008-02-29

**Authors:** Anthony Arnoldo, Jasna Curak, Saranya Kittanakom, Igor Chevelev, Vincent T. Lee, Mehdi Sahebol-Amri, Becky Koscik, Lana Ljuma, Peter J. Roy, Antonio Bedalov, Guri Giaever, Corey Nislow, Rod A. Merrill, Stephen Lory, Igor Stagljar

**Affiliations:** 1 Terrence Donnelly Centre for Cellular and Biomolecular Research, Department of Biochemistry, University of Toronto, Ontario, Canada; 2 Department of Molecular Genetics, University of Toronto, Ontario, Canada; 3 Department of Microbiology and Molecular Genetics, Harvard Medical School, Boston, Massachusetts, United States of America; 4 Clinical Research and Human Biology Divisions, Fred Hutchinson Cancer Research Center, Seattle, Washington, United States of America; 5 Department of Pharmaceutical Sciences, University of Toronto, Ontario, Canada; 6 Department of Molecular and Cellular Biology, University of Guelph, Ontario, Canada; Yale University, United States of America

## Abstract

*Pseudomonas aeruginosa* is an opportunistic human pathogen that is a key factor in the mortality of cystic fibrosis patients, and infection represents an increased threat for human health worldwide. Because resistance of *Pseudomonas aeruginosa* to antibiotics is increasing, new inhibitors of pharmacologically validated targets of this bacterium are needed. Here we demonstrate that a cell-based yeast phenotypic assay, combined with a large-scale inhibitor screen, identified small molecule inhibitors that can suppress the toxicity caused by heterologous expression of selected *Pseudomonas aeruginosa* ORFs. We identified the first small molecule inhibitor of Exoenzyme S (ExoS), a toxin involved in Type III secretion. We show that this inhibitor, exosin, modulates ExoS ADP-ribosyltransferase activity *in vitro*, suggesting the inhibition is direct. Moreover, exosin and two of its analogues display a significant protective effect against *Pseudomonas* infection *in vivo*. Furthermore, because the assay was performed in yeast, we were able to demonstrate that several yeast homologues of the known human ExoS targets are likely ADP-ribosylated by the toxin. For example, using an *in vitro* enzymatic assay, we demonstrate that yeast Ras2p is directly modified by ExoS. Lastly, by surveying a collection of yeast deletion mutants, we identified Bmh1p, a yeast homologue of the human FAS, as an ExoS cofactor, revealing that portions of the bacterial toxin mode of action are conserved from yeast to human. Taken together, our integrated cell-based, chemical-genetic approach demonstrates that such screens can augment traditional drug screening approaches and facilitate the discovery of new compounds against a broad range of human pathogens.

## Author Summary

Microbial resistance to antibiotics is a serious and growing threat to human health. Here, we used a novel approach that combines chemical and genetic perturbation of bakers yeast to find new targets that might be effective in controlling infections caused by the opportunistic human pathogen *Pseudomonas aeruginosa*. *P. aeruginosa* is the primary cause of mortality with cystic fibrosis patients and has demonstrated an alarming ability to resist antibiotics. In this study, we identified the first small molecule inhibitors of ExoS, a toxin playing a pivotal role during *P. aeruginosa* infections. One of these compounds, exosin, likely works by modulating the toxin's enzymatic activity. We further show that this inhibitor protects mammalian cells against *P. aeruginosa* infection. Finally, we used yeast functional genomics tools to identify several yeast homologues of the known human ExoS targets as possible targets for the toxin. Together, these observations validate our yeast-based approach for uncovering novel antibiotics. These compounds can be used as starting point for new therapeutic treatments, and a similar strategy could be applied to a broad range of human pathogens like viruses or parasites.

## Introduction

Microbial resistance flourishes in hospitals and community settings, and represents a major threat to human health worldwide [1],[2]. Despite the threat, drug discovery methods have failed to deliver new effective antibiotics [3]. This problem is likely to worsen because major pharmaceutical and biotech companies are withdrawing from antibacterial drug discovery [4]. To address the challenge of developing new antibiotics and managing microbial resistance, alternative strategies are needed to define and inhibit pharmacologically validated targets [5]. Several lines of evidence support the hypothesis that bakers yeast *Saccharomyces cerevisiae* can contribute during early stages of antimicrobial development. Because many essential molecular mechanisms of cells are conserved, we hypothesized that bacterial virulence proteins could act similarly in both yeast and human cells. Indeed, the study of virulence proteins in *S. cerevisiae* has proved an effective alternative and proxy for a human model of bacterial infection [6],[7],[8]. In addition, *S. cerevisiae* is well-suited for screening small molecule inhibitors to inhibit overexpressed proteins [9],[10], and to discover molecules that disrupt protein-protein interactions [11]. Finally, the arsenal of available yeast functional genomics tools provides a powerful means to study the targets and pathways modulated by drugs (reviewed in [12]). Together, these observations support the idea that compound screening in *S. cerevisiae* is a powerful tool to isolate small molecule inhibitors against potential drug targets of human pathogens.

In antibacterial drug discovery, a particular concern is the emergence of multidrug resistant strains that require several drugs for efficient disease management. This problem is exacerbated in immunocompromised patients [13]. For example, *P. aeruginosa* affects immunocompromised individuals afflicted with cystic fibrosis and is the primary Gram-negative causative agent of nosocomial infections [14]. *P. aeruginosa* is resistant to the three major classes of antibiotics, namely β-lactams, aminoglycosides and fluoroquinolones [15]. Notably, *P. aeruginosa* strains have demonstrated an alarming ability to resist antibiotics, underscoring the need to discover novel molecules with new mechanisms of action [16],[17]. Ironically, there are few innovative antibacterial molecules available or under development and the majority of these target Gram-positive bacteria [18]. Therefore, research on the opportunistic Gram-negative bacterium *P. aeruginosa* is medically relevant and is a logical choice to explore the utility of the yeast-based approach to discover new small-molecule inhibitors.

A key feature of a number of Gram-negative bacterial infection is the Type III Secretion System (T3SS) [19]. *P. aeruginosa* manipulate host cells by injecting four effector proteins, exoenzyme S (ExoS), exoenzyme T (ExoT), exoenzyme Y (ExoY) and exoenzyme U (ExoU), through the T3SS. ExoS and ExoT are bifunctional enzymes containing an amino-terminal GTPase-activating protein domain and a carboxy-terminal ADP-ribosylation domain. They inhibit phagocytosis by disrupting actin cytoskeletal rearrangement, focal adhesions and signal transduction cascades [20]. ExoY is an adenylate cyclase that elevates intracellular levels of cyclic AMP and causes actin cytoskeleton reorganization [21]. ExoU is a phospholipase whose expression correlates with acute cytotoxicity in mammalian cells [6],[22]. Therefore, targeting *P. aeruginosa* virulence factors with small molecule inhibitors would be expected to modulate the pathogen's virulence and provide a starting point for antimicrobial drug development [23].

The pivotal role played by ExoS during *P. aeruginosa* infection validates this toxin as a promising target to discover small molecules that may interfere with *P. aeruginosa* pathogenicity and infectivity [20]. ExoS was initially described as a secreted ADP-ribosyltransferase (ADPRT) [24]. The toxin is a well-characterized bi-glutamic acid transferase that requires interaction with a 14-3-3 protein (FAS) for its activity [25],[26]. Vimentin was characterized as the first direct target of ExoS ADPRT activity [27]. Shortly after, Ras and related proteins including Rab3, Rab4, Ral, Rap1A and Rap2 were identified as been modified by ExoS [28],[29]. ADP-ribosylation of Ras at arginine 41 blocks the interaction of Ras with its guanine nucleotide exchange factor (GEF), resulting in the inactivation of the Ras signal transduction pathway in the infected host cell [30]. Recently, ExoS was shown to ADP-ribosylate diverse molecules including cyclophilin A and Ezrin/Radixin/Moesin (ERM) proteins [31],[32]. During infection of HeLa and fibroblast cells, *P. aeruginosa* translocates ExoS and induces cell death by apoptosis [33],[34].

In this study, we used a novel combined phenotypic and chemical genomics screen in yeast to identify the first small molecule inhibitors of *P. aeruginosa* ExoS. The compound, exosin, modulates the toxin ADP-ribosyltransferase enzymatic activity *in vitro* suggesting the inhibition is direct. Furthermore, we observed a protective effect with this compound against *P. aeruginosa* in a well-established mammalian cell infection assay. Interestingly, although we designed the yeast phenotypic screen to assay heterologous proteins, we also observed that yeast Ras2p is directly modified by ExoS and we biochemicaly characterized this modification. This result reveals that bacterial toxins can target similar proteins in both human and yeast and validates our yeast-based approach for the study of toxin function and for the high-throughput screening for small molecule inhibitors. These initial lead compounds can be used as a starting point for new therapeutic treatments or can help to characterize the cellular functions of bacterial proteins. A similar strategy could also be applied to facilitate the discovery of new compounds against a broad range of human pathogens.

## Results

### Identification of *P. aeruginosa* Drug Targets Modulating *S. cerevisiae* Growth

**Figure 1:**
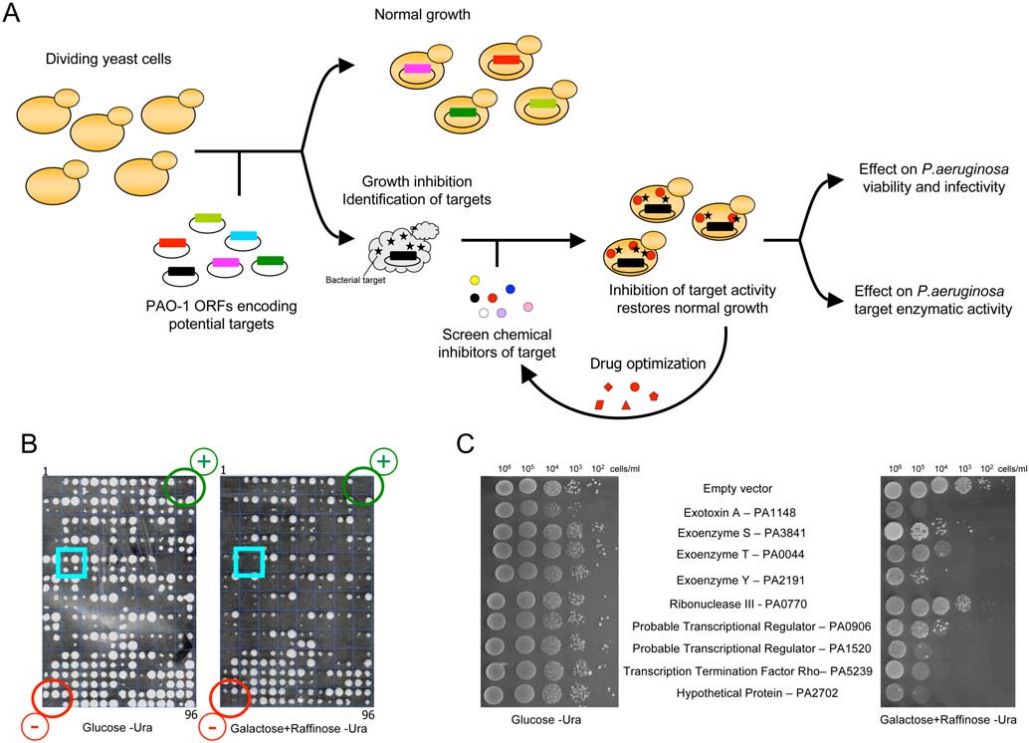
Overview of the yeast based approach to find inhibitors against the human pathogenic bacteria *P. aeruginosa* and phenotypes of the bacterial ORFs causing synthetic lethality in yeast. (A) *S. cerevisiae* W303-1A was utilized to identify *P. aeruginosa* PAO-1 virulence factors or essential ORFs that inhibit yeast growth. *P. aeruginosa* ORFs that inhibited yeast growth when individually overexpressed are prioritized based on biological relevance. Genes of interest are subsequently screened for inhibitors by overexpressing the bacterial ORFs and assaying for restoration of yeast growth in the presence of small molecules. Finally, *in vitro* and *in vivo* experiments demonstrate that the inhibitor directly modulates the bacterial protein biological activity. (B) Yeasts harboring the plasmid with the *P. aeruginosa* ORFs were grown overnight in liquid media. The cultures were then robotically diluted 10, 100 and 1000 times before their transfer in duplicate on solid media containing either glucose (Control 100% growth - left panel) or galactose + raffinose (Induction of bacterial gene expression - right panel). Cell growth was compared to the yeast with the empty vector (negative control – red circle) and to the yeast harboring the vector pRS316 encoding the toxic gene TUB2 (positive control – green circle). Phenotype of yeast with the plasmid pYES-DEST52 coding for a *Pseudomonas* toxic gene is marked by a blue square. The plasmid pYES-DEST52 was selected based on its strong promoter GAL1 combined to its high copy number 2 µ origin of replication for a maximal protein expression. (C) Nine *P. aeruginosa* ORFs inhibiting yeast growth when overexpressed. Yeast were transformed with the pYES-DEST52 yeast expression vector encoding the nine bacterial ORFs, grown overnight and individually spotted in duplicate as a 10 fold serial dilution on plates containing either glucose (left panel) or galactose + raffinose (right panel).

We developed a yeast-based strategy where *S. cerevisiae* was initially used to identify *P. aeruginosa* PAO1 virulence factors or essential ORFs that inhibit yeast growth (Figure 1A). These particular genes were selected because they provide an attractive starting point to develop antibacterial drugs. Accordingly, we developed a list of 505 potential drug targets of *P. aeruginosa* (Table S1) [35],[36],[37]. These bacterial ORFs were individually transferred into the yeast expression vector, pYES-DEST52 where the *GAL1* promoter controlled their expression. Transformed yeast growing on 2% glucose served as control (i.e., wild type growth) because in these conditions, the expression of the exogenous bacterial genes is repressed. Expression of these genes was induced by growing the yeast on selective solid medium containing 2% galactose + 2% raffinose (Figure 1B). The experiment was repeated (four times), and involved inoculating yeast cultures at different dilutions and spotting variable volumes of culture on agar plates in an attempt to increase the consistency of this test.

Of the 505 *P. aeruginosa* ORFs screened, nine strongly or partially impaired the yeast growth when overexpressed (Figure 1C). Five of these are essential genes, including; 1) the ribonuclease III – PA0770, 2) two probable transcription regulators - PA0906 and PA1520, 3) the transcription termination factor Rho – PA5239 and 4) a hypothetical protein – PA2702. In addition, four virulence genes were also detrimental to yeast growth, ExoA – PA1148, ExoS – PA3841, ExoT – PA0044, ExoY – PA2191. Interestingly, each of these four toxins are secreted or translocated by the type II (ExoA) or type III secretion system (ExoS, ExoT, ExoY) and each act within the infected host cell. By comparing the phenotype of yeast harboring the empty vector, we could assess the relative strength of the *Pseudomonas* gene overexpression effect and classify them into three groups (Figure 1C – right panel). Firstly, ExoA, ExoY, PA1520 and PA2702 strongly inhibited *S. cerevisiae* growth. Secondly, ExoS, ExoT, PA0906 and transcription termination factor Rho showed an intermediate growth impairment whereas expression of ribonuclease III weakly affected yeast fitness.

### Yeast Growth Inhibition Is Mediated by Exoa and Exos ADPRT, and Exoy Adenylate Cyclase Activities

**Figure 2:**
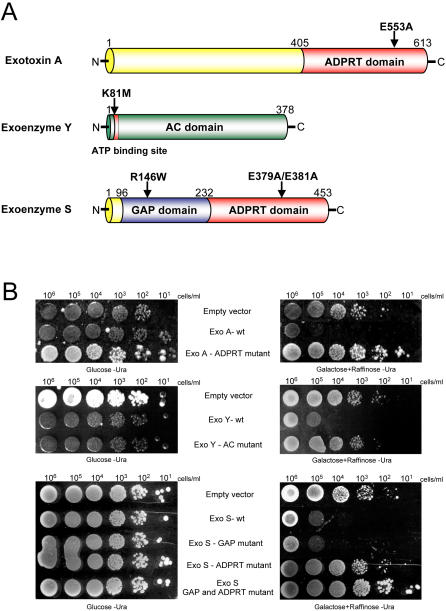
Prevention of *S. cerevisiae* growth by ExoA-ADPRT, ExoY-adenylate cyclase and ExoS-ADPRT activities. (A) Functional domains of the *Pseudomonas* ExoA, ExoY and ExoS and localizations of the point mutations abolishing the different enzymatic activities. (B) *S. cerevisiae* W303-1a was transformed with yeast expression vector alone (Empty vector), the yeast expression vector encoding ExoA wild type (ExoA wt) or ExoA E553A ADPRT mutant (ExoA-ADPRT mutant). Similarly, yeast was transformed with a vector containing ExoY wild-type (ExoY-wt), ExoY K81M AC mutant (ExoY-AC mutant). Finally, identical vector with ExoS wild type (ExoS-wt), ExoS R146W GAP mutant (ExoS-GAP mutant), ExoS E379A+E381A ADPRT mutant (ExoS-ADPRT mutant) or ExoS GAP and ADPRT double mutant (ExoS-GAP and ADPRT mutant) was incorporated in yeast. Toxicity of the different constructs in yeast were determined by spotting serial dilution of overnight cultures onto agar containing glucose (Control 100% growth - left panel) or galactose + raffinose (right panel).

To assess the influence of ExoA, ExoY and ExoS enzymatic activities on yeast growth, catalytic mutants were assayed. Residues important for the enzymatic activity of ExoA (E553A), ExoY (K81M) and ExoS (R146W and E379A+E381A) were previously reported [21],[38],[39],[40] and served to guide our mutant construction (Figure 2A).

Compared to the empty vector control, overexpression of active ExoA-wt and ExoY-wt induced a severe growth defect (Figure 2B – top and middle panels) whereas expression of the enzymatically inactive ExoA-ADPRT mutant and ExoY-AC mutant did not. This observation suggests that ExoA and ExoY toxicity is conferred by their ADPRT and AC activities, respectively. Moreover, whereas ExoS-wt expression reduced yeast growth, this dominant negative effect was totally abolished when expression of the ExoS-GAP and ExoS-ADPRT mutants were simultaneously induced, indicating one or both ExoS enzymatic activities are causative for the yeast growth defect (Figure 2B – bottom panel). Because normal growth was observed only for the ExoS ADPRT domain mutant and not for the GAP mutant, this suggests that ExoS ADPRT enzymatic activity is responsible for the yeast toxicity consistent with previous observations [41]. Taken together, these observations attribute the yeast growth inhibition to the ExoA-ADPRT, ExoY-AC and ExoS-ADPRT activities and validate the three toxins as appropriate drug target candidates for further study. Due to its critical role in the initial steps of chronic infections of immuno-compromised patients and in the pathogenesis of acute *P. aeruginosa* infections, ExoS was selected for interrogation using our yeast-cell based inhibitor screen.

### Exoenzyme S ADP-Ribosylates Identical Targets in Both Human and Yeast

**Figure 3:**
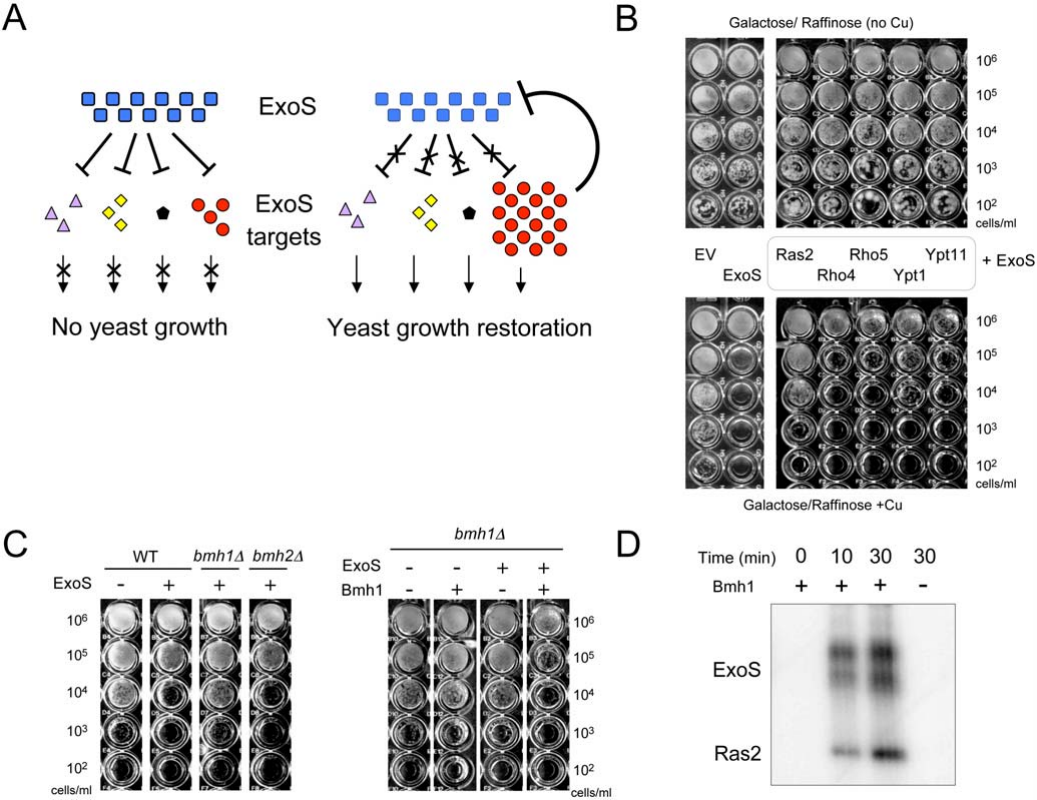
Ras is a direct target of ExoS both in yeast and human. (A) In mammalian cells, ExoS inactivates several targets. A similar mechanism could explain the observed growth defect in *S. cerevisiae*. In that case, overexpression of the yeast homologues of the ExoS human targets should restore yeast growth by titrating the toxin enzymatic activity. (B) In the presence of ExoS, yeast Ras2p overexpression reverts yeast growth to a level comparable to the one with the yeast harboring the empty vector. Overnight cultures of the yeast transformed with empty vector, the vector encoding ExoS alone or exoenzyme S with the yeast ORFs were adjusted to the same cell density (10^6^ cells/ml). The rescuing effect due to the yeast ORFs expression was estimated by spotting a 10 fold serial dilution of the yeast cultures on agar containing glucose - copper (upper panels) or galactose + raffinose + copper (lower panels). (C) Bmh1p acts as ExoS cofactor in yeast. The ExoS expression vector was transformed in wild-type, *bmh1*Δ and *bmh2*Δ yeast backgrounds. In absence of *BMH1*, but not *BMH2*, ExoS did not display any toxicity in yeast (left panel). Additionally, the vectors encoding ExoS and Bmh1p were cotransformed. Bmh1p overexpression restored ExoS toxicity in a yeast *bmh1*Δ background (right panel). (D) ExoS ADP-ribosylates Ras2p *in vitro*. Ras2p was incubated with ExoS and [^32^P]NAD in presence or absence of the yeast activator protein Bmh1. The samples were separated by SDS-PAGE and incorporation of radioactive ADP-ribose analysed by phosphorimaging. The upper bands are caused by ExoS auto-ribosylation and served as a positive control. Due to the nature of the ExoS purification, the top band corresponds to the full-length ExoS and the lower band represents the auto-ribosylation of a truncated form of ExoS.

To demonstrate that yeast can serve as a model system to mimic human cells during infection, we asked if these bacterial toxins modulate the biological activity of conserved eukaryotic targets. Following binding of *P. aeruginosa* to human cells, the bacteria inject ExoS directly into the cytoplasm where it inhibits the activity of several targets by ADP-ribosylation. Therefore, overexpressing yeast homologues of ExoS human targets should restore yeast growth by titrating the toxin's enzymatic activity (Figure 3A). To test our hypothesis, forty-six yeast members of the Ras superfamily and cyclophilins were individually overexpressed in yeast in the presence of ExoS (Table S2).

We first verified that individually, the overexpressed yeast proteins did not impair yeast growth. To accomplish this, cells were cultivated on galactose + raffinose in absence of copper such that only the yeast over-expressed candidates, but not ExoS, were expressed (Figure 3B – top right panel). Yeast genes whose overexpression was toxic were eliminated from our analysis. In parallel, cells were grown on galactose + raffinose in presence of copper to assess the rescuing effect of yeast gene overexpression in the presence of the toxic ExoS (Figure 3B – bottom right panel). Comparing yeast growth to the cell harboring the empty vector and yeast expressing ExoS alone (Figure 3B – bottom left panel), ten yeast genes were found to rescue ExoS toxicity (Table S3). Subsequently, only yeast genes demonstrating a strong growth rescue phenotype (such as *RAS2*) were analyzed further whereas genes showing weak rescue (such as *YPT1*) were not studied further (Figure 3B). *S. cerevisiae* possesses two homologues of the human Ras protein (Ras1p and Ras2p). Interestingly, Ras2p was found among these ten ORFs, i.e. overexpression of Ras2p but not Ras1p rescued ExoS-induced toxicity (Figure 3B).

As previously described, ExoS requires Factor Activating Exoenzyme S (FAS) for its ADPRT activity [26]. FAS is a member of the 14-3-3 protein family which has two yeast homologues, the Brain Modulosignalin Homolog (Bmh) 1 and 2. Accordingly, ExoS toxicity was assessed in the absence of Bmh1p or Bmh2p. As detected by the increase in yeast growth, ExoS-induced toxicity was diminished in cells lacking Bmh1p but not in those lacking Bmh2p (Figure 3C – left panel). In a *bmh1*Δ yeast background, the toxic effect of ExoS was again restored when introducing *BMH1* in the presence of the toxin (Figure 3C – right panel). Together, these data imply that Bmh1p acts as ExoS cofactor in yeast.

To better understand the mechanism of this toxicity, we demonstrated that yeast Ras2p was a direct target of ExoS and that Bmh1p was the ExoS cofactor in yeast, using a biochemical assay. To that end, an ADP-ribosyltransferase enzymatic assay was performed using the radioactive substrate [^32^P]-NAD^+^, purified *P. aeruginosa* ExoS, yeast Ras2p and Bmh1p. Autoradiographic analysis showed that radioactive ADP-ribose was incorporated by Ras2p (Figure 3D). Moreover, in absence of Bmh1p, no ADP-ribosylation was observed. These data reveal that *in vitro*, Ras2p is directly ADP-ribosylated by ExoS with Bmh1p as a cofactor.

Taken together, these results allow us to conclude the following; (i) in yeast, the growth inhibitory effect observed in the presence of the *P. aeruginosa* ExoS is mediated by its ADPRT activity, (ii) this growth inhibition is due, at least in part, to the inactivation of the yeast protein Ras2p by ADP-ribosylation, (iii) ExoS ADPRT activity is activated by the yeast cofactor Bmh1p. Most significantly, conservation of several toxin targets from yeast to human, such as Ras2p, Rsr1p, Ypt52p and Cpr6p, suggests that *P. aeruginosa* ExoS acts in a related manner in both organisms.

### E216-5303 Modulates Exoenzyme S ADPRT Activity through Competitive Inhibition

**Figure 4:**
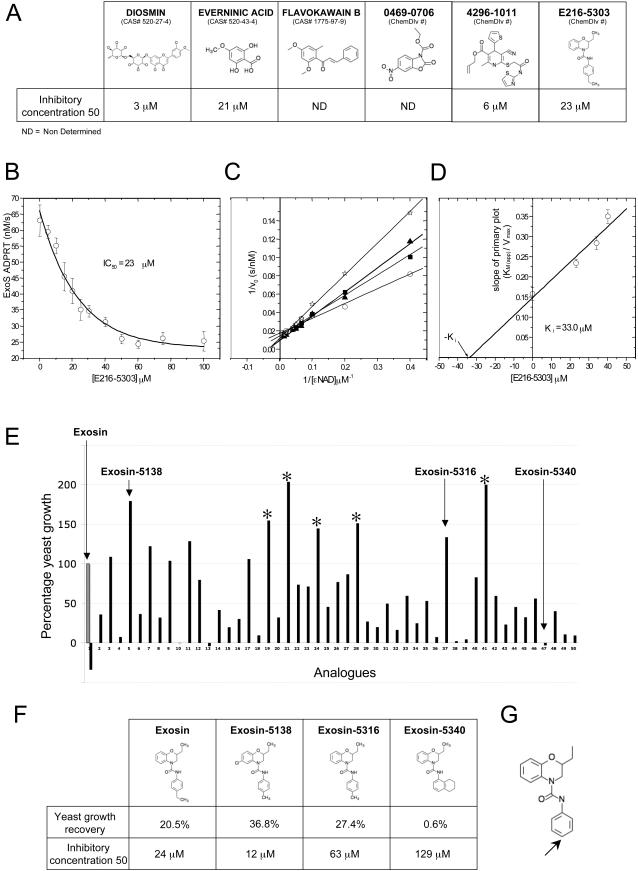
Exosin inhibits ExoS ADPRT activity. (A) List of ExoS potential inhibitors isolated during the yeast chemical screen. In yeast, the growth inhibition caused by ExoS expression was used to screen for novel inhibitors of this bacterial protein by selecting those compounds that can restore growth to the yeast expressing the toxin. Structures of the six identified hits are displayed. Diosmin, Everninic acid, 4296-1011 and E216-5303 directly modulate ExoS ADPRT enzymatic activity. IC_50_ for each molecule is defined by the compound concentration required to decrease ExoS ADPRT activity by 50%. ExoS, its cofactor FAS and human Ras were purified and used in the fluorescence-based ADPRT assay. The inhibitor IC_50_ was determined by non-linear regression curve fitting. Flavokawain B and 0469-0706 possessed intrinsic fluorescence that interfered with the fluorescent ADPRT enzymatic assay, therefore the IC_50_ for these compounds could not be determined. (B) Dose-response curve for E216-5303 on the ADPRT activity of ExoS. Various aliquots of a stock solution of E216-5303 prepared in DMSO were pre-incubated with 20 µM human Ras, 1 µM of FAS and 20 µM of ε-NAD^+^ in 100 mM NaCl, 2 mM MgCl_2_, 200 mM sodium acetate, pH 6.0. The reaction was initiated with the addition of 50 nM ExoS and the transferase reaction was monitored by recording the time-dependent change in fluorescence intensity. The fluorescence excitation was at 305 nm with fluorescence emission at 405 nm. The inhibitor IC_50_ was determined by non-linear regression curve fitting. (C) Lineweaver-Burk plot of the inhibition of ExoS ADPRT activity at 0(○), 20(▪), 30(▴) and 40(Π) µM of E216-5303. (D) Plot of the slope from (c) (K_M_/V_max_) against E216-5303 concentration (see Material and Methods for details). (E) Percentage of yeast growth induced by exosin analogues. Growth of yeast expressing ExoS was calculated for each of the 50 exosin analogues and compared to the cell expressing ExoS in the presence of exosin (100% growth control) and yeast with ExoS alone (no inhibitor – background growth control). (F) Structures, percentage of yeast growth recovery and IC_50_ for the small molecule inhibitor exosin and its analogues. Yeast growth recovery was calculated as the difference of growth of yeast expressing ExoS in the presence of the inhibitor compare to yeast harboring the empty vector (100% growth control) and yeast with ExoS alone (no inhibitor – background growth control). Exosin and analogues directly modulate ExoS ADPRT enzymatic activity. The IC_50_ values for exosin and analogues were determined as previously described. (G) Structure of the core molecule for exosin and analogues. An arrow indicates the para position of the benzyl ring, a position important for activity.

The sensitivity and specificity of our yeast-based assay allowed us to use *S. cerevisiae* to detect potential inhibitors of the three selected *P. aeruginosa* drug targets. Because ExoS-wt inhibited yeast growth when overexpressed, we reasoned that any molecule that inhibits this enzyme should restore yeast growth (Figure 1A). Because we were unable to find any inhibitors when the bacterial toxin was expressed using the strong promoter *GAL1*, we exchanged the *GAL1* promoter with the copper inducible promoter *CUP1* which allows a titrable expression of the toxins. Expression from this promoter decreases the toxin level in yeast and renders the conditions of the yeast screen less stringent. Over 56,000 compounds, primarily synthetic small molecules, were tested against ExoS. Effect of the compounds was compared to the yeast growth in absence of compound (as control for inhibition) and to the cells dividing in absence of toxin (as a control for growth). With this strategy, we uncovered six potential inhibitors, Diosmin, Everninic acid, Flavokawain B, 0469-0796, 4296-1011 and E216-5303 based on their ability to restore yeast growth (Figure 4A).

To determine if the observed yeast growth recovery was due to a direct modulation of the compounds on ExoS ADPRT activity, an *in vitro* fluorescent ADPRT enzymatic assay was performed. Diosmin, 4296-1011, Everninic acid and E216-5303 modulated ExoS ADPRT activity and their IC_50_ values were determined as 3, 6, 21 and 23 µM respectively (Figure 4A). Due to their intrinsic fluorescence, Flavokawain B and 0469-0706 effects could not be tested in our enzymatic assay. Because, only exosin protected CHO cells from lysis during *P. aeruginosa* infection in cell culture (data not shown) it was therefore selected for additional studies. Exosin acts as competitive inhibitor against the NAD^+^ substrate of ExoS as the V_max_ values were largely unaffected, whereas the K_M_ values increased from 9 to 30 µM (Figure 4C). The K_i_ value (dissociation constant for a competitive inhibitor) was 33.0 ± 3.0 µM for exosin (Figure 3D), which agrees favourably with the IC_50_ value for this compound (Figure 4B). Thus, the drug-like compound exosin directly modulates ExoS ADPRT activity *in vitro* via competitive inhibition. Therefore, exosin seems to restore ExoS dependant yeast growth defect by directly inhibiting ExoS ADPRT activity.

### Exosin Protects Exoenzyme S Induced Cytotoxicity in CHO Cells

To determine if the small molecule inhibitor, exosin, could modulate the viability of CHO cells during *P. aeruginosa* infection, apoptotic CHO cells and living CHO cells were distinguished using the exclusion dye 7-AAD. Here, CHO cells were exposed to *P. aeruginosa* with or without the small molecule inhibitor for 2 hours, and the fraction of apoptotic cells was measured by 7-AAD staining and flow cytometry.

**Figure 5:**
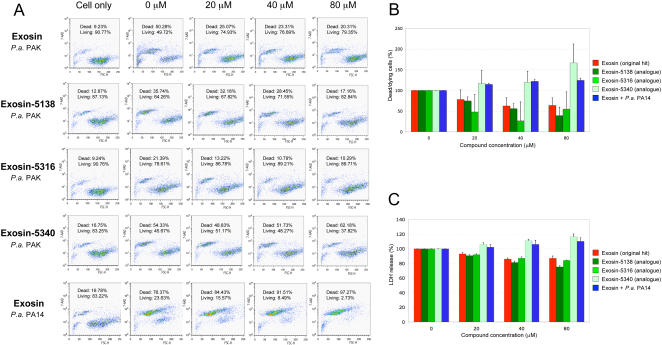
Inhibitors reduce *P. aeruginosa* cytotoxic effect during CHO cell infection. CHO cells were seeded at a concentration of 1.25×10^5^ cells/well 12 hours prior *P. aeruginosa* PAK infection (MOI 10). (A) During the early stage of infection (2 hours), CHO cells were harvested by trypsination and resuspended in HBSS + 1% BSA. Induced cell death was measured by flow cytometry after 7-AAD (10 µg/ml) staining, using a Beckman-Coulter EPICS Elite flow cytometer. Dot plots shows the percentage of dead and living cells for different compound concentrations. The lower right quadrant shows living CHO cells that are positive in size (FSC-H) and negative by 7-AAD staining. In the upper left quadrant, the 7-AAD positive location indicates the number of dead or dying CHO cells after *P.aeruginosa* infection. These results are representative of 3 independent experiments. (B) The bar graph shows the percentage of dead/dying cells measured by flow cytometry. The error bars represent the SD with n = 3. The protective effect was compared to the number of dying/dead CHO cells in absence of both bacteria and compound as the 0% of cell lysis (background control) and to the number of dying/dead CHO cells in the presence of *P. aeruginosa* but in absence of any compound as the 100% lysis. (C) During the late stage of infection (4 hours), CHO cell supernatants were submitted to LDH release quantification using the Cytotoxicity Detection Kit (Roche). The error bars represent the SD with n = 3. The percentage of LDH release for each compound concentrations was compared to the LDH release of CHO cells in absence of both bacteria and compound (spontaneous lysis) and to the LDH release of CHO cells in the presence of *P. aeruginosa* PAK in absence of inhibitor (100% lysis).

The mean fluorescent intensities of 7–AAD were plotted as a histogram (Figure S1). When compared to the mean fluorescent intensity from the red peak (background fluorescent intensity - 7.42; n = 3) and from the blue peak (control for *P. aeruginosa* infection - 18.2; n = 3), the green (20 µM), orange (40 µM), and light blue (80 µM) peaks gave mean fluorescent intensities of 13.4, 11.3, and 10.3, respectively, indicating that exosin exerted its effect in a dose-dependent manner. Therefore, a higher inhibitor dose reduced the number of cells undergoing apoptosis, reflecting a better protective effect. Similar observations were made in dot plots (Figure 5A). In the presence of 80 µM exosin, a significant increase in the percentage of living cells (79.35%; n = 3) was observed with the serious reduction of dead cells (20.31%; n = 3), compared to the infected CHO cells without inhibitor, 0 µM (49.72% and 50.28%, respectively). However, the protective effect of exosin at a concentration of 80 µM was not observed when CHO cells were infected by the *P. aeruginosa* PA14 strain, a strain expressing ExoT, ExoY and the phospholipase exoenzyme U (ExoU) but not ExoS, indicating the specificity of the compound exosin against ExoS only (Figure 5A – lower panels).

In the CHO cell infection assay, the protective effect of exosin was monitored during an early stage of infection by detecting the number of dying and dead CHO cells using flow cytometry. Moreover, the effect of the inhibitor at the late stage of infection was assessed by the quantification of lactate dehydrogenase (LDH) released from the population of lysed CHO cells. Four hours after *P. aeruginosa* PAK infection (Figure 5C) revealed a 6.93% decrease in lysis upon addition of 20 µM exosin, a 13.92% lysis reduction in the presence of 40 µM final of inhibitor and a 12.90% reduction at 80 µM. However, the protective effect of exosin at a concentration of 80 µM was not observed when CHO cells were infected by the *P. aeruginosa* PA14 strain that translocates ExoU instead of ExoS (Figure 5C). Together, these data strongly support the conclusion that the inhibitor exosin is specific for ExoS and is able to reduce ExoS cytotoxicity against mammalian cells.

*P. aeruginosa* PAK viability was tested by measuring optical density of cultures in the presence of 20, 40 and 80 µM of inhibitor over a period of 10 hours. Addition of exosin did not affect *Pseudomonas* growth, further confirming the specificity of exosin for ExoS in CHO cells (Figure S2).

### Pre-Selection of Exosin Analogues in *S. cerevisiae*

Given the specificity of exosin, we screened 50 structural analogues of this compound in yeast to find molecules with increased potency against ExoS ADPRT activity. Seven analogues with an improved effect were found (Figure 4E – exosin-5138, exosin-5316 and the compounds marked by an asterisk). According to the flow cytometry results, all of these compounds protected CHO cells when infected with *P. aeruginosa* in cell culture (data not shown). However, only exosin-5138 and exosin-5316 showed a protective effect when monitored with the LDH assay. Therefore, only these two compounds were used for further investigation. Exosin-5340 had no protective effect in yeast or in the CHO cell infection assay and served as a negative control. Importantly, results obtained from the yeast studies revealed the importance of the para position of the nitrobenzyl ring for the inhibitory activity of the compounds (Figure 4G). The three different analogues, exosin-5138, exosin-5316 and exosin-5340, were then selected for quantification of the yeast growth recovery and for IC_50_ determination. Exosin-5138 showed 36.8% recovery, almost double the protective effect of exosin whereas exosin-5340 conferred no protection (Figure 4F). The last analogue exosin-5316 (with 27.4% recovery) demonstrated a protective effect almost equal to the original compound (20.5% recovery). The fluorescent ADPRT enzymatic assay revealed that the three analogues directly modulate ExoS ADPRT activity *in vitro* (Figure 4F). The IC_50_ for each compound was calculated and these values paralleled the effect of the small molecules in yeast, most strongly for exosin-5138 and exosin-5340, and to a lesser extent for exosin-5316.

We extended our studies of these analogs in mammalian cells. For this purpose, protection provided by exosin-5138 and exosin-5316 was assessed in the CHO cell toxicity assay. Using the flow cytometry as described earlier, a strong protective effect of exosin-5138 was observed (Figure 5A – left panel). Dot plots of exosin-5138 showed a large reduction in dead cells at a compound concentration of 80 µM (Figure 5A – right panel). Exosin-5138 showed decreases of 25.20, 44.05 and 60.83% in the number of dead/dying CHO cells in the presence of 20, 40 and 80 µM of inhibitor, respectively (Figure 5B). In the LDH assay, exosin-5138 reduced cell lysis by 9.34, 18.61 and 24.64% in presence of the compound at 20, 40 and 80 µM inhibitor, respectively (Figure 5C) demonstrating an improved efficacy of exosin-5138 against ExoS cytotoxicity versus the original hit.

In addition, as shown by flow cytometry, exosin-5316 exerted a protective effect (Figure 5A). The number of dead/dying CHO cells was detectably lower upon addition of the analogue exosin-5316; however, this reduction was not statistically significant compared to the original hit (p>0.05). In contrast, the LDH assay revealed a 7.94, 12.84 and 15.81% reduction in cell lysis at 20, 40 and 80 µM final concentration respectively (p<0.05). The analogue exosin-5316 showed similar protective effect compared the original hit (p<0.05) (Figure 5C).

The data show a correlation between the protective effect of exosin and its analogues in yeast and for the results obtained in the CHO cell infection assay. Moreover, these observations establish yeast as a powerful assay system to estimate the effect of analogues of an original hit and to prioritize lead compounds before tedious subsequent experiments in a more complicated model of infection are undertaken.

## Discussion

In this report, we used the *P. aeruginosa* virulence factor, ExoS, to demonstrate the utility of the baker yeast *S. cerevisiae* as a tool to isolate inhibitors against human pathogens. We succeeded in identifying the first known inhibitor of ExoS, exosin, and demonstrated that this assay can be used to uncover structural analogs with improved potency. Therefore, yeast can substitute for traditional human models of infection, and be used to effectively prioritize compounds.

### Expression of *P. aeruginosa* Genes Affecting Yeast Growth

In our report, *S. cerevisiae* produced a binary readout that allowed us to test 505 *P. aeruginosa* genes for their inhibitory effect in yeast. Expression of nine bacterial genes, five essential and four virulence genes, reproducibly prevented *S. cerevisiae* growth. Among the isolated essential genes, the Rho termination factor from *E. coli* was demonstrated to induce yeast RNA polymerase II release at all pause sites of the mRNA *in vitro* [42]. Thus, transcription deregulation could explain the yeast growth arrest in the presence of the transcription termination factor Rho. Members of the ribonuclease III superfamily are RNA-specific endonucleases involved in RNA maturation, RNA degradation and gene silencing [43]. We hypothesize that the observed yeast growth defect induced by expression of the *P. aeruginosa* ribonuclease III was caused by deregulated RNA degradation. Since no clear biological function is associated with the two probable transcriptional regulators – PA0906 and PA1520, nor for the hypothetical protein – PA2702, we cannot speculate on the mechanism of action of these proteins in yeast.

During infection, *P. aeruginosa* manipulates host cellular function through the action of the toxins ExoA, ExoS, ExoT and ExoY using the type II and III secretion systems [44]. Since these toxins inactivate key molecules directly within the infected cells and because several basic molecular functions are conserved among eukaryotes, it seemed likely that the toxins could act similarly on targets conserved in both yeast and human. Therefore, inactivation of yeast homologues of the toxin human targets is an attractive and simple scenario to explain the growth inhibition effect conferred by the ExoA, ExoS, ExoT and ExoY expression in yeast. There are many possible reasons to account for the limited number of bacterial genes affecting yeast growth including; (i) a proper expression of the bacterial gene (e.g. high CG content in the *Pseudomonas* DNA sequence [45]), (ii) the presence of the target and/or appropriate co-factor and (iii) required post-translational modifications.

### Conservation of ExoS Biological Activity in Both Human and Yeast

High conservation of basic molecular and cellular mechanisms between yeast and human cells highlights *S. cerevisiae* as an ideal model organism to study mammalian diseases and their underlying pathways [46],[47]. Indeed, several reports have shown that this conservation can be used to decipher bacterial toxins mode of action [6],[7],[48]. Here, the toxicity caused by ExoA, ExoY and ExoS overexpression in yeast is mediated by their enzymatic activity (Figure 2B). Interestingly, ExoS ADPRT activity alone is sufficient to induce yeast cell growth arrest. This last observation inspired us to study ExoS yeast toxicity into more detail.

Once translocated in human host cells via *P. aeruginosa* type III secretion system, ExoS inhibits several cellular targets. Its GTPase activating protein activity reorganizes the actin cytoskeleton through RhoA, Rac1 and Cdc42 inactivation. In contrast, ExoS ADPRT activity inactivates a wide range of proteins such as several members of the Ras family and related proteins, cyclophilin A and the Ezrin/Radixin/Moesin (ERM) proteins. Since ExoS inactivates all its protein targets, overexpression of the yeast homologues of the known human targets should restore yeast growth by titrating ExoS enzymatic activity. The ERM proteins play a role during cell polarity establishment of multi-cellular organisms and no homologues were found in yeast. Therefore, only yeast members on the Ras superfamily and cyclophilin family were induced in our overexpression study. Globally, no yeast homologue of the human RhoA, Rac1 or Cdc42 rescued ExoS-induced toxicity indicating that, together with the results obtained in the ExoS mutagenesis experiments, the ExoS GAP activity does not play a role in ExoS yeast growth inhibition. Four homologues of the human ExoS targets Ras, Rap1b, hRab and CyclophilinA were identified as the yeast Ras2p, Rsr1p, Ypt52p and Cpr6p, respectively. Arf1p, a member of the Sar/Arf family and homologue of the human Arf1 protein, rescued yeast expressing ExoS. Arf1p is involved in the retrograde transport of vesicles from the trans Golgi to the plasma membrane. Biological relevance of Arf1p as a direct target of ExoS is questionable since, during *P. aeruginosa* infection, ExoS trafficking occurs from the plasma membrane to the perinuclear region [49]. In our system, ExoS is encoded by a plasmid, thus deregulation of the retrograde transport could prevent ExoS reaching its plasma membrane targets (e.g. Ras2) and explain why Arf1p overexpression allowed yeast to divide in the presence of ExoS. No homologues of Cin4p (Sar/Arf family), Cns1p, Cpr7p, Cpr8p exist in human confounding the interpretation of these results.

ExoS overexpression in the absence of Bmh1p, the yeast homologue of the ExoS cofactor FAS, was not toxic to yeast (Figure 3C). *In vitro* enzymatic assay demonstrated that ExoS ADP-ribosylated Ras2p with Bmh1p as a cofactor (Figure 3B). Interestingly, substitution of the yeast Ras2p by the constitutively active Ras2-G19V mutation did not prevent ExoS toxicity (data not shown). Additionally, previous results showed that a deletion mutant of ExoS (lacking aa 51–72 membrane localization domain), which cannot ADP-ribosylate Rasp *in vivo*, is nevertheless as cytotoxic as wild type ExoS in CHO cells, indicating that Ras ADP-ribosylation is dispensable for ExoS virulence [50]. These observations suggest that, in yeast, the observed growth inhibition may be due to the cumulative inhibitory effects of ExoS on already known targets and/or due to the additional inactivation of an unknown key protein(s).

### Yeast as a Tool for Drug Screening and Prioritization

ExoS plays a pivotal role in the establishment of *P. aeruginosa* chronic infections and during acute *P. aeruginosa* pathogenesis. For that reason, this toxin was selected in our yeast phenotypic system to find inhibitors capable of modulating its enzymatic activity. Here, we report the isolation of exosin, the first inhibitor of ExoS ADPRT activity using a yeast cell-based screen. Six compounds were isolated from a library of 56,000 compounds and all rescued ExoS induced toxicity in yeast (Figure 4A). This relatively small number of hits is likely due to several reasons; both chemical and biological. The compound library, though selected for its diversity, nonetheless samples a limited range of chemical space. Furthermore, of 6,000 compounds that were randomly picked from the library and tested against ExoS and ExoY in two different yeast genetic backgrounds (the wild-type and the *pdr1*Δ+*pdr3*Δ strains), no difference in potency was observed, suggesting that the yeast genotype did not substantially influence the number of hits obtained in our screen.

In the enzymatic and the CHO cells infection assays, only exosin modulated ExoS biological activity. The apparent inactivity of the five other small molecules can likely be explained by two arguments. First, the five inhibitors could exert their effect on molecules or pathways modulated by ExoS without directly inhibiting ExoS ADPRT activity. Furthermore, because these five compounds exerted no protective effect in the CHO cell infection assay we predict they act on yeast specific pathways. A second explanation could be that the effect observed in yeast requires metabolism of the compound and is not an effect of the original compound itself.

Our results revealed that compounds derived from natural products were more bioactive on yeast (3 primary hits out of 580 natural products). However, the only hit conferring protection during infection of CHO cells by *P. aeruginosa* belongs to the class of the drug-like synthetic compounds (3 primary hits out of 53,000 compounds). Thus, there is certainly much more chemical space that can be probed using both natural and synthetic compounds.

### E216-5303 Mode of Action

Exosin was shown to directly interact with ExoS *in vitro* as a competitive inhibitor against the NAD^+^ substrate of ExoS ADPRT activity with comparable IC_50_ and K_i_ values indicating that exosin likely binds to the NAD^+^-pocket within the ADPRT domain of ExoS. This was substantiated by our observation of a similar inhibitory effect of exosin on the ADPRT activity of ExoA (IC_50_  =  17 µM; data not shown). Unfortunately, no high-resolution structure has been determined for the ADPRT domain of ExoS; however, ExoA is a well-characterized ADPRT enzyme for which there is a crystal structure of the ADPRT catalytic domain in complex with substrates and inhibitors [51],[52],[53]. By analogy with the recent X-ray co-crystal structure of ExoA with PJ34 [51], the benzylmorpholine ring of exosin might be expected to intercalate into the nicotinamide pocket within ExoS. In this scenario, the inhibitor amide should form an H-bond with enzyme. Presently, the site of contact within the ExoS active site for the alkyl group on the exosin nitrobenzyl moiety (shown by an arrow in Figure 4G) is not known; however, a single ring is required for *in vivo* activity and an ethyl is preferred over a methyl at the alkylation site. In summary, although we lack atomic resolution, it appears likely that the *in vivo* inhibitory activity of exosin against ExoS toxicity is due to a direct interaction of the inhibitor with the ADPRT domain of the toxin.

### Conclusion

Using a yeast cell-based screen, the first known inhibitor of the *P. aeruginosa* ExoS, called exosin, was isolated and several analogues of the original hit were characterized. This work was facilitated by the partial conservation of the proteins inactivated by ExoS in both human and yeast. Thus, *S. cerevisiae* is a powerful tool to study bacterial toxins and to identify their corresponding inhibitors. Future studies could extend a similar approach to a broad range of human pathogens such as viruses and bacteria.

## Materials and Methods

### 

#### Strains

*S.cerevisiae* W303-1A (*MATa his3 ade2 leu2 trp1 ura3 can1*) and 14328-pdr1+pdr3 (*Mat*α *his3 met15 ura3 pdr1::Nat pdr3::KanMX*) were propagated at 30°C on yeast–peptone–dextrose (YPD) or synthetic dextrose (SD) minimal medium missing the appropriate amino-acid. *P. aeruginosa* strains PAK [54] and PA14 [55] were routinely grown at 37°C in Luria-Bertani broth.

#### Constructs

The 505 ORFs coding the *Pseudomonas aeruginosa* PAO1 drug targets, contained in the entry vector pDONR201 [56], were individually subcloned in the yeast expression vector pYES-DEST52 (Invitrogen) by LR reaction according to the manufacturer's instruction (Invitrogen). Proper integration of the ORFs in pYES-DEST52 was checked by PCR using the forward primer pDONR201-F (5′-TCGCGTTAACGCTAGCATGGATCTC-3′) and reverse primer pDONR201-R (5′-GTAACATCAGAGATTTTGAGACAC-3′).

ExoA-E553D enzymatic mutant was PCR amplified from *Pseudomonas aeruginosa* genomic DNA using the primers pYES-DEST52-top (5′- ACAAGTTTGTACAAAAAAGCAGGCTCCGAAGGAGATACCATGCACCTGACACCCCATTGGATCC-3′) with ExoA-E553D-Bot (5′-GGCCAGCCGAGAATGGTGTCCAGGCGCCCGCCTTCC-3′) and ExoA-E553D-Top (5′-GGAAGGCGGGCGCCTGGACACCATTCTCGGCTGGCC-3′) with pYES-DEST52-Bot (5′-ACATGATGCGGCCCTCTAGGATCAGCGGGTTTAAACTCAATGGTGATGGTGATGATGACCGG-3′). The different PCR products were subsequently recombinationally cloned into pYES-DEST52 (Invitrogen).

ExoS-R146K enzymatic mutant was PCR amplified from *Pseudomonas aeruginosa* genomic DNA using the primers ExoS-top-ivrec (5′- TCGGATCCACTAGTAACGGCCGCCAGTGTGCTGGAATTATGCATATTCAATCGCTTCAGC-3′) with ExoS-R146K-Bot (5′-CCAAGGCGGTGCTCAGCGACTTCAGCGCCCCATCTCCGCTGG-3′) and ExoS-R146K-Top (5′- CCAGCGGAGATGGGGCGCTGAAGTCGCTGAGCACCGCCTTGG-3′) with ExoS-bot-ivrec (5′- ATTACATGATGCGGCCCTCTAGATGCATGCTCGAGCGGCCTCAGGCCAGATCAAGGCC-3′). The different PCR products were subsequently recombinationally cloned into pYES2 (Invitrogen).

ExoS-E379A+E381A enzymatic mutant was PCR amplified from *Pseudomonas aeruginosa* genomic DNA using the primers ExoS-top-ivrec (5′- TCGGATCCACTAGTAACGGCCGCCAGTGTGCTGGAATTATGCATATTCAATCGCTTCAGC-3′) with ExoS-E379A+E381A-Bot (5′- CGGTCTCTTTGTTATAGAGAATCTCTTTTTCATTCTTGTAGTTCGATATCCCGC -3′) and ExoS-E379A+E381A-Top (5′- GCGGGATATCGAACTACAAGAATGAAAAAGAGATTCTCTATAACAAAGAGACCG-3′) with ExoS-bot-ivrec (5′- ATTACATGATGCGGCCCTCTAGATGCATGCTCGAGCGGCCTCAGGCCAGATCAAGGCC-3′). The different PCR products were subsequently recombinationally cloned into pYES2 (Invitrogen).

ExoS-R146+E379A+E381A double mutant was PCR amplified from *Pseudomonas aeruginosa* genomic DNA using the primers ExoS-top-ivrec (5′- TCGGATCCACTAGTAACGGCCGCCAGTGTGCTGGAATTATGCATATTCAATCGCTTCAGC-3′) with ExoS-R146K-Bot (5′-CCAAGGCGGTGCTCAGCGACTTCAGCGCCCCATCTCCGCTGG-3′), ExoS-R146K-Top (5′- CCAGCGGAGATGGGGCGCTGAAGTCGCTGAGCACCGCCTTGG-3′) with ExoS-E379A+E381A-Bot (5′- CGGTCTCTTTGTTATAGAGAATCTCTTTTTCATTCTTGTAGTTCGATATCCCGC -3′) and ExoS-E379A+E381A-Top (5′- GCTAACAAAGAGACCG-3′) with ExoS-bot-ivrec (5′- ATTACATGATGCGGCCCTCTAGATGCATGCTCGAGCGGCCTGGGATATCGAACTACAAGAATGAAAAAGAGATTCTCTACAGGCCAGATCAAGGCC-3′). The different PCR products were subsequently recombinationally cloned into pYES2 (Invitrogen).

ExoY-K81M enzymatic mutant was PCR amplified from *Pseudomonas aeruginosa* genomic DNA using the primers ExoY-topivrec (5′-TCGGATCCACTAGTAACGGCCGCCAGTGTGCTGGAATTATGCGTATCGACGGTCATCGTC-3′) with ExoY-K81M-Bot (5′-CCCCTTCACCGAGAAGCCCATGGTCGGGAAACCC-3′) and ExoY-K81M-top (5′-GGGTTTCCCGACCATGGGCTTCTCGGTGAAGGGG-3′). The different PCR products were subsequently recombinationally cloned into pYES2 (Invitrogen).

ExoA, ExoS and ExoY was PCR amplified from *Pseudomonas aeruginosa* genomic DNA and recombinationally cloned into pDH105 using the oligonucleotides PA1148-pDH105ivrec-Top (5′-AGGCAAGATAAACGAAGGCAAAGGACGGTTCTAGAGCTGACATGCACCTGACACCCCATTGGATCC-3′) and Pa1148-pDH105ivrec-Bot (5′- CACACAGGAAACAGCTATGACCATGATTACGCCAAGCTTCTGCAGTTACTTCAGGTCCTCGCGCGGCGG-3′) for ExoA, ExoS-pDH105ivrec-Top (5′AGGCAAGATAAACGAAGGCAAAGGACGGTTCTAGAGCTGAC ATGCATATTCAATCGCTTCAGCAGAG-3′) and ExoS-pDH105ivrec-Bot (CACACAGGAAACAGCTATGACCATGATTACGCCAAGCTTCTGCAGTCAGGCCAGATCAAGGCCGCG) for ExoS,ExoY-pDH105ivrec-Top (5′- AGGCAAGATAAACGAAGGCAAAGGACGGTTCTAGAGCTGAC ATGCGTATCGACGGTCATCGTCAG-3′) and ExoY-pDH105ivrec-Bot (5′- CACACAGGAAACAGCTATGACCATGATTACGCCAAGCTTCTGCAGTCAGACCTTACGTTGGAAAAAGTCGAG-3′) for ExoY.

The plasmids pEGH [57] for yeast ORFs overexpression were kindly provided by R. Sopko.

### *Pseudomonas aeruginosa* ORF Screening

*S. cerevisiae* strain W303-1A harboring the plasmid pYES-DEST52 coding each of the 505 *Pseudomonas aeruginosa* PAO1 drug targets was grown overnight in SD–Ura to maintain selection of the plasmid. Yeast culture was directly submitted to 3 steps of a ten fold dilution using the liquid handling robot Q-Bot (Genetix). The non-diluted and diluted cultures were then immediately inoculated in duplicate on solid medium containing either glucose-Ura (repressing conditions) or galactose+raffinose-Ura (inducing conditions). Plates were incubated at 30°C and monitored for yeast growth defect after 2 and 3 days. Growth was compare to the fitness of yeast harboring the empty vector and to the yeast harboring the toxic pRS316-TUB2 vector [58].

### Compound Screening

The LOPAC library (1,280 compounds, Sigma-Aldrich), the SPECTRUM library (2,000 compounds, MicroSource Discovery Inc.) and a ChemDiv library (53,000 compounds) were screened at a final concentration of 50 µM. *S. cerevisiae* strain 14328-pdr1+pdr3 harboring the plasmid pDH105-exoA, pDH105-exoS or pDH105-exoY was grown overnight in SD -Leu to maintain selection of the plasmid and were diluted to a cell density of 5×10^3^ cells/ml. Addition of 0.9 mM (ExoA and ExoY) or 1.5 mM (ExoA) of CuSO_4_ induced the expression of the toxin in yeast, the cultures were then aliquoted into wells of 96-well plates and compounds were added. Plates were incubated at 30°C and inspected for yeast growth recovery after 24 and 48 hours. As a control, cells containing the empty vector pDH105 were similarly grown, diluted and inoculated with copper and 0.5% DMSO. The effect of the hits on yeast growth recovery was quantified as a percentage of growth as described elsewhere [10].

### CHO Cells Toxicity Assay

Chinese hamster ovary (CHO) cells toxicity assay was performed as previously described with minor modifications [59]. CHO cells were routinely grown in F-12 medium supplemented with 10% fetal bovine serum (FBS) and 2 mM glutamine. Prior to infection, confluent CHO cells were washed and incubated with F-12 containing 1% FBS and 2 mM glutamine. *P. aeruginosa* was grown overnight in LB, subcultured into fresh LB, and grown to mid-log phase. 2.5×10^5^ CHO cells per well were infected with *P. aeruginosa* at an initial multiplicity of infection (MOI) of 10 in duplicate. After 2 hours infection, CHO cells were harvested by trypsination and resuspended in Hank's Balanced Salt Solution + 1% bovine serum albumin. Induced cell death was measured by flow cytometry after 7-AminoActinomycin D (10 µg/ml) staining, using a Beckman-Coulter EPICS Elite flow cytometer. Culture supernatants from a second duplicate of CHO cells were collected after 4 hours of infection and centrifuged for 10 min at 3,220×g to sediment bacteria and CHO cells. Lactose dehydrogenase (LDH) in the supernatant was measured with a Roche LDH kit in accordance with the manufacturer's instructions. Percent LDH release was calculated relative to that of the uninfected control, which was set at 0% LDH release, and that of cells lysed in absence of inhibitor, which was set at 100% LDH release.

### Protein Purifications

Recombinant yeast FAS, human Ras, yeast Ras2p, yeast Bmh1p and Bmh2p were cloned into pEGX (GE Healthcare), transformed in *E. coli* BL21 and purified according to manufacturer's instructions. Recombinant ExoS was purified by gel filtration and ion exchange chromatography as previously described [60].

### *In vitro* ADPRT Enzymatic Assay

To monitor incorporation of ADP-ribose into yeast Ras2, 50 nM of purified ExoS was added to a 20 µl reaction mixtures containing 1 µM of Bmh1, 20 µM of yeast Ras2, 2 mM of MgCl_2_ and 200 mM of sodium acetate (pH 6.0). The reaction was initiated by adding 2 Ci of ExoS radioactive substrate, nicotinamide adenine [adenylate-^32^P] dinucleotide (^32^P-NAD) (1000 Ci/mmol, GE Healthcare). Reaction mixes were incubated for 0 to 30 min at 30°C. The reaction was terminated with 2× Laemmli sample buffer, resolved by a 15% SDS-PAGE, and analyzed by autoradiography using a Typhoon Trio Workstation (GE Healthcare). The auto-ADP-ribosylation of ExoS served as a positive control in each reaction and reaction missing FAS served as a negative control.

### Kinetic Analysis of ExoS ADPRT Activity

The NAD^+^-dependent ADPRT assay of ExoS was performed at 30°C with ExoS at 50 nM in the presence of 1 µM of FAS and 20 µM human Ras in 100 mM NaCl, 2 mM MgCl_2_, 200 mM sodium acetate, pH 6.0 while the concentration of ε-NAD^+^ varied between 0 and 75 µM. The reaction was initiated with the addition of 50 nM (final conc.) ExoS in an Ultravette (Brand Scientific) 70 µL cuvette and the transferase reaction was monitored by recording the time-dependent change in fluorescence intensity with a PTI AlphaScan-2 fluorimeter (PTI Inc., New Jersey) with 305 nm and 405 nm excitation and emission, respectively. The data were analyzed by nonlinear curve fitting using the Michaelis-Menten equation (OriginLab v6.1; Northampton, MA) and also by linear regression analysis of both the Hanes-Woolf and the Lineweaver-Burk (LB) plots. The K_i_ values were determined for various inhibitor compounds (1.4% DMSO, final conc.) using Dixon plots, as well as from secondary plots of the slope of the LB plots versus inhibitor concentration. IC_50_ values, the concentration of the inhibitor that reduces the activity of the enzyme by 50%, were determined by non-linear regression curve fitting using Origin 6.1 [51].

## Supporting Information

Figure S1 Inhibitors reduce P. aeruginosa cytotoxic effect during CHO cell infection. CHO cells were infected by P. aeruginosa PAK or PA14 (MOI 10) as described in Figure 5A. Mean fluorescence of the total CHO cells was calculated for the inhibitors as represented in the histograms.
(101.02 MB TIF)

Figure S2 Exosin does not affect P. aeruginosa PAK viability. P. aeruginosa PAK overnight culture was inoculated at a cell density of 2.5×104 or 2.5×105 cells/ml, and grown in the presence of 20, 40 and 80 µM of inhibitor exosin. Growth curves were calculated for bacteria dividing in the presence of DMSO (control for growth), of the antibiotics Penicillin/Streptomycin (control for growth inhibition) or exosin over a period of 10 hours. These results are representative of 3 independent experiments.
(101.02 MB TIF)

Table S1 List of the tested P. aeruginosa essential genes (PA number) and tested P. aeruginosa virulence genes (PA number)
(0.07 MB XLS)

Table S2 RAS superfamily and Cyclophilins
(0.04 MB XLS)

Table S3 Yeast genes
(0.03 MB XLS)
