# Correction: Identification of Small Molecule Inhibitors of *Pseudomonas aeruginosa* Exoenzyme S Using a Yeast Phenotypic Screen

**DOI:** 10.1371/annotation/76d35829-07a2-479f-bbc1-cce6755b6d8c

**Published:** 2008-04-02

**Authors:** Anthony Arnoldo, Jasna Curak, Saranya Kittanakom, Igor Chevelev, Vincent T. Lee, Mehdi Sahebol-Amri, Becky Koscik, Lana Ljuma, Peter J. Roy, Antonio Bedalov, Guri Giaever, Corey Nislow, A. Rod Merrill, Stephen Lory, Igor Stagljar

The first name and middle initial of the 13th author were switched. The correct name is A. Rod Merrill. In the Author Contributions section, Igor Stagljar (IS) should be listed as one of the persons who wrote the paper.

